# When familiarity not novelty motivates information-seeking behaviour

**DOI:** 10.1038/s41598-023-31953-6

**Published:** 2023-03-30

**Authors:** Gregory Brooks, Hannah Whitehead, Stefan Kӧhler

**Affiliations:** 1 Graduate Program in Neuroscience, Western University, London, N6A 3K7 Canada; 2 Department of Psychology, Western University, London, N6A 3K7 Canada; 3 Ontario Institute for Studies in Education, University of Toronto, Toronto, M5S 1V6 Canada

**Keywords:** Psychology, Human behaviour, Motivation

## Abstract

Research has established that novelty motivates information-seeking behaviour in many situations. While novelty preferences have been well-studied, an understanding of conditions under which familiarity trumps novelty remains limited. Recent work has revealed that when a metacognitive experience indicates that unsuccessfully recalled information may still be available, a subsequent tendency to seek out unrecalled familiar information can emerge. We conducted three experiments to identify critical factors that determine when familiarity preferences can be observed. Experiment 1 demonstrated the critical role of a recent unsuccessful recall attempt in inducing such a preference. Experiment 2 revealed that the impact of recall attempts is not limited to situations that follow unsuccessful recall, as a familiarity preference was observed even when information was successfully generated. Experiment 3 showed that the level of confidence in the accuracy of any recalled information is a key factor, with moderate levels of confidence leading to the strongest subsequent familiarity preference. Together, our results suggest that novelty preferences in information-seeking are not ubiquitous, as specific situational demands including recent attempted memory retrieval, as well as metacognitive retrieval experiences, can induce familiarity preferences. Our findings can be interpreted within theoretical frameworks that emphasize the role of knowledge gaps as driving factors of information-seeking.

## Introduction

In daily life we have the potential to be exposed to a vast amount of information. The question of how we decide which information to consume has garnered the interest of researchers in a range of disciplines, including psychology and neuroscience. In some circumstances, we will seek out instrumental information that we can strategically use to guide future behaviour. Notably, however, at other times we consume non-instrumental information that has no direct benefit to us. To illustrate, consider the recent times that you have checked your Twitter feed. This act is an example of information-seeking. If your Twitter feed shows that there has been an accident on your route to work, you will be able to adjust your behaviour accordingly, in order to avoid delays. In this case, the information you viewed would be instrumental. Also likely, however, is you choosing to view a Tweet regarding the love life of a celebrity, which has no targeted use in informing specific future behaviour, making it non-instrumental. State curiosity is defined as the intrinsic motivational factor that drives non-instrumental information-seeking behaviour^1–4^.

Extant research has provided considerable evidence speaking to the behavioural and experiential consequences of state curiosity. Studies have revealed that the satiation (or resolution) of curiosity provides an intrinsic sense of satisfaction in which sought out information acts as reward (e.g. Refs.^5–7^). A considerable literature (for a review see^8^) has also demonstrated that when curiosity is resolved information that one was curious about is more likely to be subsequently remembered than information that one was not curious about. These studies have often employed trivia questions to induce curiosity and have demonstrated memory enhancement for the answers to questions that were associated with high levels of curiosity, as well as for unrelated information encountered in close temporal proximity to them^9–11^. In neuroscientific research, extant work has demonstrated links between curiosity and areas of the brain that are well-known to be related to reward processing (e.g. Ref.^12^), such as the ventral tegmental area and nucleus accumbens^9,10^. While much is known about the consequences of state curiosity, as summarised in broad strokes here, there has been considerably less research on its potential sources and their relationship to memory processing.

A dimension of particular interest when aiming to understand sources of state curiosity is the novelty versus familiarity continuum. A large body of research points to close ties between novelty, curiosity, and exploratory behaviour (e.g. Refs.^13,14^). However, there have also been a few demonstrations suggesting that, under some circumstances, curiosity is tied to the exploration of familiar information that has previously been encountered (e.g. Refs.^15,16^). The question of what conditions lead to information-seeking behaviour that is geared towards such familiar information is the focus of the set of experiments we conducted in the present study.

### Novelty versus familiarity preferences

Novelty preferences in information-seeking have been linked to states of curiosity that diminish with further stimulus exposure^13^. This large body of work suggests that the more novel an aspect of the environment is, the greater the level of curiosity and likelihood of engagement in information-seeking behaviour. Supporting evidence has come from pioneering studies by Berlyne^1^ demonstrating that rodents, when given one novel stimulus alongside two items with which they had previous exposure, spend more time exploring the novel stimulus. Similar evidence for novelty preferences in exploration in humans has emerged from the visual-paired comparison (VPC) task, often used in infant memory research^17^. In this paradigm, one novel stimulus and one to which participants were introduced previously, are shown side-by-side, while visual fixation patterns are examined as a marker of curiosity. A central result from numerous studies is that infant and adult populations spend more time fixating on novel items compared to familiar ones^18,19^ (see below for exceptions).

Findings with the VPC task pose an important question as to the cause of novelty preferences. One suggestion within the literature is that novelty preferences carry adaptive value; that is, a preference for novelty can lead to the reduction of uncertainty. As novelty is typically associated with uncertainty, the preference to explore a new environment can lead to the discovery of new information that can, in turn, be used to make more beneficial future decisions^14^. In line with this general idea, benefits of novelty for learning have also been reported. Such learning benefits are most prevalent under experimental conditions in which novelty is defined as a violation of expectations derived from a given situational context (for review see Refs.^20,21^). Pertinent evidence comes from findings showing, for example, that when multiple similar stimuli are presented, a stimulus that differs from the rest is more likely to be subsequently remembered^22^.

While early views of curiosity were ubiquitous in their emphasis of novelty as a driver for information-seeking (e.g. Ref.^13^), recent work has indicated that in some circumstances, curiosity may be directed towards familiar stimuli, even in the VPC task paradigm^15^. Richmond and colleagues^15^ employed face stimuli in an experiment in which they varied the length of the delay between the familiarisation and test phase of the VPC task, and examined how delays affect novelty preferences, as measured by fixation duration. They reported novelty preferences with delays of up to 2-weeks, no preferences with delays of 6-weeks, and familiarity preferences after delays of 12-months. The researchers argue that this shift in preference from novelty towards familiarity is reflective of the accessibility of a memory. Indeed, these researchers’ follow-up experiment, in which participants provided explicit judgements of familiarity, confirmed that the accessibility of information about prior occurrence, as reflected in both accuracy and response times, decreased over time. Their research contributes to a larger body of research suggesting that novelty preferences are not ubiquitous in exploration on VPC tasks^23,24^.

Critical evidence in support of the notion that novelty is not a ubiquitous driver of curiosity also stems from work that has examined exploratory behaviour following unsuccessful memory recall. Specifically, this research has addressed how metacognitive retrieval experiences during unsuccessful recall may shape subsequent information-seeking behaviour. A metacognitive phenomenon that has been of particular interest in this context is the feeling-of-knowing (FOK) experience, which represents the feeling that we might be able to recognize an answer that we cannot recall among multiple alternatives^25^. Recent work from our lab suggests that FOK experiences can induce a subsequent preference for the familiar information that could not be recalled, as compared to entirely novel information, in exploration^16^. In this study a modified FOK paradigm was used, which involved a study phase for face-name pairs, a FOK test phase, and a subsequent exploration phase. In the FOK phase participants were shown a mix of previously studied and new faces and were asked to recall the associated name and provide a FOK judgement (“How likely is it that you could recognize this person’s name?”). In the exploration phase, participants could choose up to half of the presented faces for exploration of the associated names. A key component of this paradigm was the inclusion of new faces, for which the associated names had not been studied, in the FOK and exploration phases. This set-up allowed us to test for the presence of novelty or familiarity preferences under conditions of limited exploration opportunities. We demonstrated that the names for faces that induced higher FOKs were sought more frequently than names for faces with lower FOKs. Critically, results also showed that information-seeking was directed more frequently towards the exploration of previously studied names that could not be successfully recalled than novel ones that had never been encountered. This pattern of results poses important new questions as to the exact nature of retrieval conditions that induce a subsequent preference for familiarity over novelty in exploration.

### Retrieval factors that may induce a subsequent familiarity preference

While demonstrating a familiarity preference in information-seeking, the study by Brooks et al.^16^ was not designed to identify the specific demands of memory tasks and the scope of metacognitive retrieval experiences that may lead to this subsequent preference in exploration. From a theoretical perspective it is important to consider the influential work of Loewenstein^2^ that proposes an important link between information gaps and curiosity. Loewenstein suggests that states of curiosity reflect the desire to gain knowledge that contributes to the minimization of information gaps. This holds relevance for FOKs, as these experiences surface when information cannot be recalled, and an information gap is perceived to be present.

A more recent theoretical approach that extends this work is known as the Region-of-Proximal Learning (RPL) theory^26–29^. It suggests that curiosity peaks when an information gap is of a specific size. This optimal information gap is proposed to be the range in which it is small enough to be judged as possible to be closed. From this perspective, a FOK state reflects a metacognitive experience that provides an estimate of the size of such an information gap. A critical task factor within this framework that is implicated in typical FOK paradigms, including the one employed by Brooks et al.^16^, is the unsuccessful recall attempt that leads to these metacognitive experiences. At present it remains unclear, however, whether a targeted recall attempt, and the perceived lack of success in its outcome, are necessary conditions for eliciting subsequent familiarity preferences in information-seeking behaviour. Furthermore, it remains unknown whether retrieval experiences other than FOK states may generate similar effects on information-seeking.

An additional task factor to consider when understanding the impact of FOK experiences on subsequent information-seeking behaviour is their prospective nature. To provide judgements about FOK experiences, a participant must make predictions about future performance, a feature that distinguishes them from other metacognitive retrieval assessments that are retrospective in nature, such as probing the degree of familiarity or of confidence experienced during recognition-memory judgements. Consideration of the predictive nature of FOK judgements is important in light of findings derived from the educational literature. Specifically, there is evidence indicating that the act of making predictions about the filling of information gaps can increase curiosity, by virtue of directing attention to them^30–33^. Although the exact nature of predictions made in these studies differs from that in FOK judgements, this research suggests that their generation may have an influence on information-seeking behaviour.

### Current study

In the current study, we conducted a set of three experiments to identify critical retrieval factors of memory tasks that induce a familiarity preference in subsequent information-seeking behaviour. Towards this end, we addressed the role of (i) recent explicit recall requirements, (ii) their perceived success, and (iii) corresponding metacognitive retrieval experiences in the development of such preferences. In addition, we considered whether making predictions about future performance at the time of retrieval plays an important role. To answer these questions, we employed a three-phase experimental paradigm similar to the one employed by Brooks et al.^16^. It involved an initial study phase for face-name associations, a retrieval phase for the learnt associations, as well as a subsequent exploration phase for the names associated with previously studied and novel faces, which allowed us to probe the degree of familiarity preference expressed in information-seeking behaviour.

## Experiment 1

**Figure 1:**
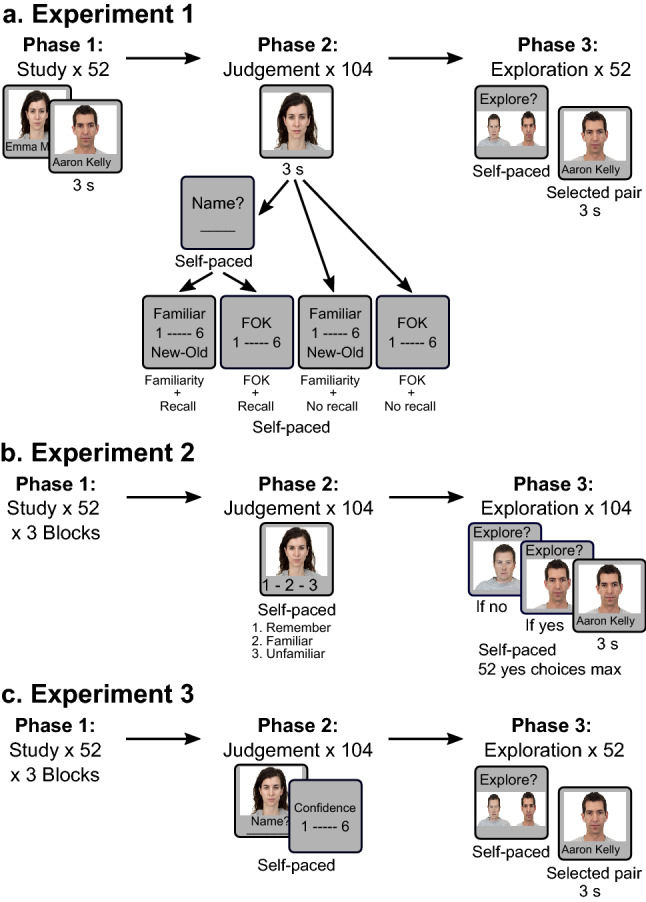
Behavioural paradigms. (**a**) Phases 1 to 3 in Experiment 1. Participants first encoded face-name pairs. Participants were assigned to one of four phase 2 conditions. The first dimension was either with an explicit recall attempt or without a recall attempt. Second, they were asked to provide either a prospective FOK judgement or a retroactive familiarity judgement. After this, they were allowed to seek a subset of names for cued faces (an information-seeking set-up also used in Experiment 3). This phase was a VPC-inspired set-up, where participants had to directly choose between viewing previously studied and new names. Following this, but not shown in this figure, was the final forced-choice recognition-memory test. (**b**) In Experiment 2, participants first studied the face-name pairs in 3 study blocks. Next, in phase 2, they provided a judgement for each face that was either: Remember, Familiar or Unfamiliar. To finish the experiment, participants were allowed to seek the name for a maximum of half the faces. They went through each face and either chose to see the name (in which case they were shown the face and name together) or chose not to see the name (progressing to the next trial). (**c**) Experiment 3, also featured 3 study blocks for phase 1, before a phase 2 that included a typed recall response, where participants were instructed to always enter something, followed by a subjective confidence rating. The experiment finished with an exploration-based phase 3, where they made information-seeking choices in the VPC-style set-up employed in Experiment 1.

In Experiment 1, we manipulated whether the memory task in the retrieval phase had an explicit recall requirement for the names of previously studied faces, and whether the memory judgement was prospective or retrospective in nature (Fig. 1a). For the prospective task, we administered FOK judgements similar to those employed in our prior work^16^, which necessitate a prediction about future performance. For the retrospective task, we chose familiarity-based recognition-memory judgements on the same faces, without any demand for predictions. Thus, the study involved a two-by-two between-subject experimental design in which half of the participants engaged in an explicit recall attempt prior to either FOK or familiarity judgements, while the rest did not. If making a prediction is key to inducing a subsequent familiarity preference, we would expect to see a main effect of judgement type in the pattern of choices for information-seeking, with FOK judgements being associated with a stronger familiarity preference than familiarity judgements. If an unsuccessful recall attempt is critical, we would expect to see an increased familiarity preference for both judgement types with the inclusion of an explicit recall requirement. We also anticipated that, if such a familiarity preference can be observed following both types of judgements, metacognitive retrieval experiences might predict subsequent choices in information-seeking for both types of judgements. To test these predictions, we probed preferences in information-seeking with a task set-up that closely resembled the one typically used in the VPC task, in which familiar information is directly pitted against novel information in regard to behavioural choices. Thus, during the exploration phase, participants were asked to choose between a face for which the name had previously been studied and a novel one for which it had not.

## Methods

### Participants

135 English-speaking participants from the online recruitment platform Prolific (https://www.prolific.co/) participated in Experiment 1 in exchange for monetary compensation. The data of 113 participants (age range 18–35; *M* = 25.92, *SD* = 5.22) were used in all analyses. 14 participants were excluded due to an insufficient distribution of FOK and familiarity ratings across the scale (i.e. less than 5 instances for 2 of the 6 scale values on all trials), and 8 participants were excluded due to failed quality control measures. These quality control criteria were introduced to ensure the data collected from online participants was comparable in quality to data collected during in-person studies. These criteria included the exclusion of participants whose response time on any catch trial was greater than 10 s, and the exclusion of those whose breaks between phases were longer than 200 s. The targeted sample size in this experiment, and the others that follow, was guided by our previous work where robust effects were observed in samples of 25 participants.

The study was approved by the Western University’s Non-Medical Research Ethics Board. Informed consent was acquired from each participant before the start of each experiment. Participants were provided monetary compensation. All experiments were performed in accordance with the approved guidelines and regulations.

### Materials

All face stimuli used in this paradigm were taken from the Chicago Face Database^34^ and were screened using the published norming data to ensure uniformity in terms of neutral emotional expression and perceived attractiveness. Selection criteria included a rating below 3.5 (on a 7-point scale) on all emotional expressions (afraid, angry, happy, sad, surprised, disgusted, and threatening), and attractiveness ratings between 2 and 5 on the 7-point scale. Of the faces that met these criteria, a total of 104 faces were randomly selected for experimental use. This database provides permission to incorporate a subset of their stimuli into published figures and have obtained consent from its participants for this usage.

For this study, English names were selected from the U.S. Census Bureau 1990 (https://catalog.data.gov/dataset/names-from-census-1990) for use in the study and recognition phases of the experiment. The total set was composed of 104 male first names, 104 female first names, and 208 surnames of medium frequency in the population (frequency rates between 0.15 and 0.50% for first names, and between 0.05 and 0.50% for surnames, respectively). Explicit efforts were made to avoid any overlap in pronunciation or spelling between the names selected (e.g. Julie and Julia or Robert and Roberts), and to avoid any reference to celebrities. First and last names were then paired to create 208 different full names of comparable length (11 to 13 characters; *M* = 12.00, *SD* = 0.80), and comparable syllable count (3 to 5). For each participant, 52 of the names were pseudo-randomly paired (on the basis of being matched for sex) with 52 of the faces for use in the study phase, and 52 were paired with the remaining 52 faces for use as novel information that could be sought in the exploration phase. The remaining 104 names were used as entirely new lures in the final forced-choice recognition test.

### Procedure

The behavioural paradigm for the current study was designed in PsychoPy3 (https://www.psychopy.org), using the Builder tool^35^, and was administered online through Pavlovia (https://pavlovia.org/). Participants were restricted to using computers to complete the study; however there was no specified screen size. The structure of Experiment 1 (Fig. 1a) included a study phase, a memory-judgement phase, an exploration phase, and a final forced-choice recognition test. Overall, the experiment took approximately 40 min to complete.

In the first part, participants were asked to memorise a set of 52 face-name pairs. Each pair appeared on the screen for 3 s with the face appearing above the name. Following a 500 ms ISI, the next pair was presented. Participants were offered a break halfway through this study phase.

Following this first phase, participants completed a second phase with memory-judgements. Prior to the experiment they were randomly assigned to one of four conditions. In two of these conditions, participants began each trial by being asked to engage in a self-paced recall attempt, which required them to type any part of the associated name for a face cue that they were able to remember, before progressing to a memory-judgement prompt. In the other two conditions, participants proceeded directly to the memory-judgement aspect of the phase. The memory-judgement was either a FOK judgement (i.e. an estimation of the likelihood that they would be able to recognize the name associated with the face prompt, if provided) or a familiarity judgement about the face cues themselves. Both of these ratings involved a 6-point Likert scale from 1 to 6 (FOK judgement: 1/‘very unlikely' to 6/‘very likely'; familiarity judgement: 1/‘sure it is new’ to 6/‘sure it is old’). Thus, the four conditions we employed were FOK with no recall, FOK with recall, familiarity with no recall and familiarity with recall. Both the FOK and familiarity judgements were self-paced and were followed by a 500 ms ISI before the start of the subsequent trial. Critically, half (52) of the face cues in this phase had been studied initially, with the rest serving as novel items.

To probe whether there was a familiarity preference present, we directly examined whether participants preferred to seek familiar or novel information. The set-up of this exploration phase was inspired by the design of the VPC task outlined in the introduction (e.g. Ref.^17^). Unbeknownst to the participants, we presented a face that had been studied initially alongside a face that had not been studied and asked them to select which associated name they most wanted to see. In other words, they were asked to make a choice about whether they wanted to be exposed to familiar or novel information. Unlike the typical VPC task in which novelty is defined in terms of the stimulus itself, novelty in our task was defined with respect to the association between the face and name. This is because all faces had been previously encountered in the memory-judgement phase, but the corresponding names were only shown for those pairs presented in the study phase. The face cues shown during this phase were matched on demographic, and the familiar face was randomly shown on the left or the right side, to control for a potential location preference. Whichever face they selected would then appear with the associated name for 3 s, followed by a 500 ms ISI before the beginning of the next trial. This phase proceeded through 52 trials, where all 52 initially studied and all 52 novel faces were shown side-by-side. No explicit mention of an additional future memory test was made before or during the exploration phase.

In a fourth and final phase of the experiment, participants completed a self-paced forced-choice recognition-memory test for the names of all 104 faces used in the memory-judgement phase (not shown in Fig. 1a). In this recognition-memory test, three name options were presented for each face, namely the name corresponding to the face, a previously seen name that belonged to one of the other previously studied faces, and an entirely new name. The three choices were matched for sex and were presented randomly in one of three positions on the screen. This type of recognition-memory test is often included in FOK research to demonstrate the validity of the subjective FOK judgments provided by participants. Due to the difficulty in interpreting results on this task when an additional exploration phase is introduced after provision of FOK judgments analyses of data from this phase were only included in the Supplementary Materials.

In each phase, catch trials were pseudo-randomly included to ensure participants were engaging in the task. In these catch trials (which participants were reminded of prior to the start of each phase), participants had to respond to a blue square as quickly as possible with a button press that was not used for any other judgement. These trials randomly occurred once every 52 trials (i.e. one catch trial in each half of the memory-judgement phase).

## Results

### Initial validation of FOK and familiarity ratings

Studies using FOK judgements often confirm the general validity in these ratings by demonstrating that they are significantly associated with subsequent accuracy in recognition-memory judgments (e.g. Ref.^36^). However, with the present set-up that involved an opportunity for further selective re-exposure to non-recalled information in the subsequent exploration phase, assessing validity in this manner becomes challenging (see Ref.^16^; nevertheless, pertinent results are included in the Supplementary Materials). Therefore, to assess the validity of participants' FOK and familiarity judgements, we primarily compared the ratings provided for previously studied and new faces, in conditions with and without explicit recall requirements. Note that we only assessed, through Bonferroni-corrected t-tests, whether the ratings differed between old and new items within each condition, without comparisons across conditions, given the different heuristic cues that can contribute to the judgements in each condition^37^.

Results showed that there was a significant difference between FOK ratings for old (*M* = 2.55, *SD* = 0.79) and new items (*M* = 1.93, *SD* = 0.71) in the FOK with recall condition,* t*(27) = 5.73, *p* < 0.001, *d* = 1.08. Old items were also rated higher (*M* = 2.91, *SD* = 0.69) than new items (*M* = 2.23, *SD* = 0.62) in the FOK no recall attempt condition, *t*(27) = 7.67,* p* < 0.001, *d* = 1.45. As FOKs are expected to be increased only for faces for which corresponding names were previously encountered, this pattern provides indirect support for validity in the judgments expressed by participants.

Likewise, familiarity ratings were significantly higher for old items (*M* = 4.07, *SD* = 0.65) than new items (*M* = 3.05, *SD* = 0.98) in the familiarity with recall condition, *t*(27) = 6.32, *p* < 0.001, *d* = 1.19. Ratings for old items (*M* = 3.84, *SD* = 0.51) were also higher than those for new items (*M* = 2.82, *SD* = 0.62) in the familiarity without recall condition, *t*(27) = 7.38, *p* < 0.001, *d* = 1.39. This pattern of results suggest that the familiarity ratings obtained also carried validity in both task conditions. Although the average ratings for entirely new and previously studied items were not close to the end points of the scale, they showed a significant difference in the expected direction, with means on different sides of the midpoint of the scale.

### Influence of retrieval-task demands on familiarity preferences in subsequent information-seeking

**Figure 2:**
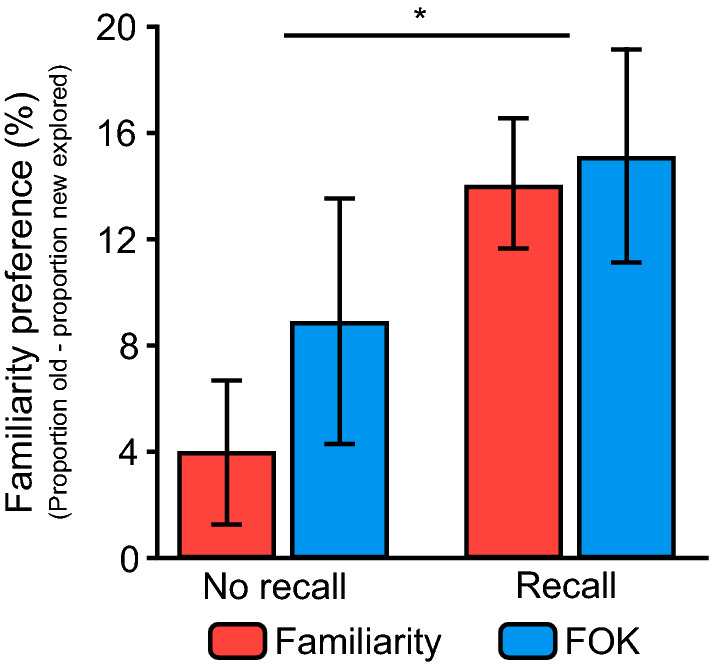
Familiarity preference in Experiment 1. The familiarity preference (as a percentage) increased in conditions involving a preceding explicit recall attempt, but there was no difference between memory judgement types, nor an interaction. Error bars =  ± 1 SEM. *p < 0.05.

We next addressed one of our two main questions of interest in this experiment; we investigated whether the requirement of an explicit recall attempt or the nature of the retrieval task (i.e. whether it is prospective or retrospective) were factors associated with an increase in familiarity preferences in subsequent information-seeking. We computed a familiarity preference for each participant by calculating the difference in frequency of exploration choices for previously studied versus novel faces (see Supplementary Materials for the frequency of exploration for old and new trials). To compare conditions with recall and those with no recall, we calculated this preference across all trials in each condition. Note that on the large majority of trials of conditions in which a recall attempt was required, this attempt turned out to be unsuccessful (FOK with recall: *M* = 98.01%, *SD* = 2.48%; familiarity with recall: *M* = 96.98%, *SD* = 2.68%). For statistical comparison of experimental conditions, we conducted a two-way ANOVA. There was a significant main effect of recall, where the average familiarity preference was higher in conditions with an explicit recall requirement (*M* = 14.56, *SD* = 17.57) compared to those without this requirement (*M* = 6.39, *SD* = 19.83), *F*(1, 108) = 5.28, *p* = 0.02, η_p_^2^ = 0.05. However, there was no significant effect of judgement type (FOK: *M* = 11.95, *SD* = 22.90; Familiarity: *M* = 8.10, *SD* = 14.40), *F*(1, 108) = 0.69, *p* = 0.41, η_p_^2^ = 0.006, nor was there a significant interaction between recall and judgement type, *F*(1, 108) = 0.27, *p* = 0.60, η_p_^2^ = 0.003 (Fig. 2). Levene’s test for inequality of variances was significant, *F*(3, 108) = 5.07, *p* = 0.003, suggesting results be interpreted with caution.

Given that variances were unequal, we also conducted non-parametric analyses to confirm the results of our ANOVA. A Mann–Whitney test confirmed that the familiarity preference was significantly greater for participants in recall conditions (*Mdn* = 13.46) than those in no recall conditions (*Mdn* = 3.84), *U* = 1142, *n*_1_ = *n*_2_ = 56, *p* = 0.01. We then compared familiarity preferences for the two judgement types; a Mann–Whitney test indicated that there was no significant difference in familiarity preferences between the FOK conditions (*Mdn* = 9.62) and the familiarity conditions (*Mdn* = 7.69), *U* = 1423, *n*_1_ = *n*_2_ = 56, *p* = 0.40. Taken together, these findings illustrate that it is the presence of a failed explicit recall attempt, rather than the prospective nature of memory judgements, which is driving subsequent information-seeking behaviour towards familiar items.

We recognize that FOKs are retrieval experiences that are typically assumed to accompany unsuccessful recall, suggesting that a recall attempt may be an inherent judgment component even if the experimental task has no explicit recall requirement. As such, we conducted an analysis calculating the RTs for FOK judgements under conditions with no recall requirement (i.e. the time between face stimulus offset and FOK response) and compared them with RTs for trials in which a recall attempt was required but was unsuccessful. For the latter set of trials we specifically focused on the period between offset of the face stimulus and the no-success response in the recall component of the judgements. We found that average RTs for this recall component were significantly longer (*M* = 1972 ms, *SD* = 1198 ms) than those for the completion of the entire FOK judgments under conditions in which there was no explicit recall requirement (*M* = 1116 ms, *SD* = 1181 ms), *t*(54) = 2.69, *p* = 0.009, *d* = 0.72. This pattern of results suggests that search times during recall attempts were longer when such attempts were explicitly demanded by the task at hand than when spontaneously included as part of the FOK judgments. It is compatible with an account that emphasizes increased cognitive effort brought on by the explicit recall requirement as a potential explanation for any related impact on subsequent information-seeking in the FOK conditions.

### Influence of retrieval experiences on subsequent information-seeking

**Figure 3:**
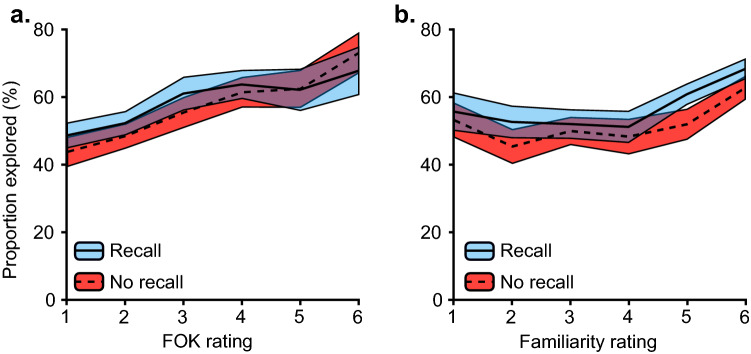
Relationships between memory-judgements and information-seeking under task conditions with or without recall requirement. In Experiment 1, the degree of FOK (**a**) and familiarity (**b**) judgements within each condition correlate with subsequent information-seeking for previously studied items, where information associated with higher FOKs or higher subjective familiarity was sought more frequently. Shaded area =  ± 1 SEM.

Finally, we calculated the relationship between the judgement ratings and subsequent information-seeking choices within each of the four experimental conditions (Fig. 3). Given that our focus was on familiarity preferences, in combination with the fact that new items had limited variance in both FOK and familiarity ratings, we examined this relationship for previously studied items only.

In the FOK with recall condition, we found that the average gamma correlation between FOK ratings and information-seeking choices (*Mean γ* = 0.16, *SD* = 0.26) was significantly above zero*, t*(27) = 3.27, *p* < 0.001, *d* = 0.62. Participants in the FOK with no recall condition also exhibited a significantly positive average gamma correlation (*Mean γ* = 0.18, *SD* = 0.27), *t*(27) = 3.54,* p* = 0.02, *d* = 0.67. The familiarity with recall condition (*Mean γ* = 0.17, *SD* = 0.24) and the familiarity with no recall condition (*Mean γ* = 0.14, *SD* = 0.24), both exhibited positive relationships, *t*(27) = 3.64, *p* = 0.001, *d* = 0.69 and* t*(27) = 2.99, *p* = 0.006, *d* = 0.56, respectively. Overall, there were no differences in the average correlations across conditions, as a two-way ANOVA conducted using the gamma correlations revealed no significant main effects (Recall: *F*(1, 108) = 0.01, *p* = 0.91; Judgement type: *F*(1, 108) = 0.16, *p* = 0.69), nor a significant interaction, *F*(1, 108) = 0.29, *p* = 0.59. In other words, there was a comparable positive relationship between ratings of retrieval experiences and information-seeking behaviour for all conditions. We also note that when we conducted these analyses focusing on all trials (i.e. regardless of objective old-new status), a very similar pattern of results emerged (see Supplementary Materials).

In a further extension of this set of analyses we also examined whether the observed relationships between retrieval experience and subsequent information-seeking are strictly linear in nature, or whether there may be a significant quadratic component. To address this issue, we fit the following equation to the data from individual participants Eq. (1):

1 $$proportion\, sought= {b}_{0}+{b}_{1}\times x+{b}_{2}\times x\times (1-x)$$

where $x=familiarity\, rating$, and conducted inferential statistics on these parameter estimates extracted from these fitted equations. This methodology resembles that described by Kang^9^ and colleagues and Dubey and Griffiths^38^. For the familiarity with recall condition, the curve fit provided an average ${r}^{2}$ of 0.49 (*SD* = 0.27). The linear (${mean\, b}_{1}$ = 10.52, *SD* = 26.80) and quadratic (${mean\, b}_{2}$ = − 47.11, *SD* = 115) coefficients were both significant, *t*(27) = 2.08, *p* = 0.05, *d* = 0.39 and *t*(27) = − 2.17, *p* = 0.04, *d* = − 0.41. For the familiarity with no recall condition, the fit provided an average ${r}^{2}$ of 0.39 (*SD* = 0.28). The linear coefficient (${b}_{1}$ = 10.79, *SD* = 30.30) showed a significant trend, *t*(27) = 1.88, *p* = 0.07, *d* = 0.36, and again the quadratic coefficient (${b}_{2}$ = − 34.99, *SD* = 83.4) was significant, *t*(27) = − 2.22, *t* = 0.04, *d* = − 0.42.

We also fit these equations to the FOK data ($x=FOK\, rating$) to explore the relationships between memory judgments and exploration, beyond our previous focus on a linear association. In the FOK with recall condition (${mean\,r}^{2}$ = 0.57, *SD* = 0.27) there was a significant linear coefficient (${b}_{1}$ = 27.14, *SD* = 40.7), *t*(27) = 3.53, *p* = 0.002, *d* = 0.67, but the quadratic coefficient was not significant (${b}_{2}$ = − 6.71, *SD* = 116, *t*(27) = − 0.31, *p* = 0.76, *d* = − 0.06). Likewise, in the FOK with no recall condition (${mean\, r}^{2}$ = 0.59, *SD* = 0.24) the linear coefficient was significant (${b}_{1}$ = 27.23, *SD* = 40), *t*(27) = 3.60, *p* = 0.001, *d* = 0.68, but the quadratic coefficient was not (${b}_{2}$ = 1.02, *SD* = 108), *t*(27) = 0.05, *p* = 0.96, *d* = 0.009.

Together, these results indicate that information-seeking peaks when an item is highly familiar, with numerically highest exploration rates for items with highest perceived familiarity. At the same time, the significant quadratic component also revealed increases for faces with extreme lack of perceived familiarity (i.e. perceived item novelty). This U-shape relationship was not present in the FOK conditions, in which curiosity increased with the degree of FOK experiences in a linear manner. Our results extend previously reported associations between metacognitive retrieval experiences and subsequent information-seeking behaviour (e.g. Ref.^16^). Here, we demonstrate that such links to information-seeking are not limited to FOK experiences and also apply to the subjectively experienced degree of familiarity with the stimulus itself, albeit with a more complex relationship.

## Experiment 2

While the results of Experiment 1 point to the importance of recall attempts as a factor of retrieval tasks that increase subsequent familiarity preferences, they do not speak to whether this increased preference is present only in situations in which recall was unsuccessful. With the paradigm employed in Experiment 1, we were unable to examine trials associated with successful recall, as there was a very limited number of these trials (due to the large number of face-name pairs and the short exposure period for each of them in the study phase). According to the RPL theory^28,29^, unsuccessful recall induces curiosity if the underlying metacognitive experience indicates that the information gap is small enough to be closed with additional information. A strong FOK experience accompanying an unsuccessful recall attempt, would indicate that the information, while currently not accessible, is in a range where it could easily be identified for closure of the gap. In line with this reasoning, one might predict that familiarity preferences in information-seeking would critically depend on the subjective perception that the recall attempt was unsuccessful. It is important to consider, however, that participants may also seek out information for reasons other than getting access to content that could not be accessed during recall. Notably, they may also use such an opportunity for gaining feedback on the accuracy of information they generated during a recall attempt. This would be in line with the general notion that curiosity serves to reduce uncertainty, and with specific findings suggesting that uncertainty about one's answers to knowledge questions can drive curiosity^9,39^. From this perspective one might expect to observe a familiarity preference in information-seeking even after recall attempts that are characterised by perceived success in generating pertinent information. In Experiment 2, we tested these ideas using a retrieval task that included metacognitive assessments of perceived recall success.

We employed multiple study blocks (Fig. 1b) to boost the learning of face-name associations and, in turn, increase the probability of subsequent recall success. To measure perceived recall success, we used a modified version of Tulving’s metacognitive Remember-Know procedure^40^. Participants could indicate one of three options in response to each face cue: that they could recall the name (“Remember” trials), that they could not recall the name despite the face feeling familiar (“Familiar” trials), or that they could not recall the name nor perceive the face as familiar (“Unfamiliar” trials). If familiarity preferences in information-seeking are critically dependent on perceived lack of success in recall, we would predict that names associated with Familiar trials would be chosen more frequently in the subsequent exploration phase than names associated with Remember trials. Alternatively, if familiar information is also sought out so as to obtain feedback for the monitoring of recall accuracy, we might expect that items from Familiar and Remember trials would be explored at comparable frequencies.

## Methods

### Participants

The online recruitment platform Prolific (https://prolific.co/) was used to recruit 78 English-speaking participants to participate in Experiment 2 in exchange for monetary compensation. To ensure there was variation in response types in the memory-judgement phase, and considering we planned to analyse the subsequent information-seeking choices in relation to these judgements, we only included participants who offered at least 5 trials of each response. With this criterion, we had to exclude 15 participants, all of whom had fewer than 5 Remember trials. We excluded an additional 4 participants who did not meet the quality control requirements outlined in Experiment 1. The data of the remaining 59 participants (age range 18–35; *M* = 24.60, *SD* = 5.00) were included in all analyses.

### Materials

The stimulus materials and randomization procedure for Experiment 2 were the same as those used in Experiment 1. Unlike in the prior experiment, however, there was no forced-choice recognition-memory test after the exploration phase, and, as such, only 104 of the names previously used were employed (i.e. the 104 new lure names were not used).

### Procedure

Experiment 2 involved a paradigm similar to that in Experiment 1 except for the following changes. First, exposure to items in the study phase was repeated 3 times (i.e. each familiar face-name pair was studied 3 times). This modification was introduced in order to increase the likelihood of recall success. Additionally, given this experiment did not require FOK judgments about future performance, there was no final forced-choice recognition-memory test. Overall, the experiment took approximately 30 min to complete.

The most significant procedural change in Experiment 2 (Fig. 1b) pertained to the response structure of the memory-judgement phase. In this phase, participants were shown faces, both old and novel, and were asked to try and recall the associated name. They were instructed to provide a self-paced judgement similar in format to a Remember/Know task. Specifically, for each item, participants could indicate one of three options: ‘I remember the name’ (Remember), ‘I don’t remember the name, but the face is familiar’ (Familiar) or ‘I don’t remember the name and the face is not familiar’ (Unfamiliar). Note that, unlike in Experiment 1, no typed recall response was required in this phase, with the consequence that objective recall accuracy could not be assessed. In other words, we rely on the self-reported recall success (via the Remember response) and refer to these trials as having perceived recall success. The next trial began after a 500 ms ISI. As in Experiment 1, this phase included all 52 initially studied faces, and 52 novel items. After completion, participants progressed to the exploration phase.

Unlike in Experiment 1, the exploration phase followed the structure employed by Brooks et al.^16^, with 104 faces presented one at a time. Participants were given an opportunity to select up to 52 of these faces in order to gain exposure to the associated names. All 104 faces had been presented previously as cues in the memory-judgement phase, but associated names had been encountered in the study phase for only 52 of them. This critical task feature allowed for estimation of any preference for familiar versus novel information. If the participant chose to see the name for a given face prompt, the face-name pair would appear on the screen for 3 s. After this interval, or if they chose not to see the name, the next face would appear, following a 500 ms ISI. Throughout this phase, participants were also shown a countdown of how many more face-name pairs were still available for exposure. If the participant reached the maximum of 52 possible exposures, they were forced to choose ‘no’ for the remaining information-seeking choices. No explicit mention of an additional future memory test was made before or during the exploration phase.

## Results

### Initial validation of responses on modified Remember-Know task

To assess whether our study manipulation was successful in Experiment 2, we focused on the distribution of responses in the memory-judgement phase for previously studied and new items (Supplementary Table S1). We expected that the previously studied faces would produce increased name recall and feelings of familiarity relative to faces that had not been seen. A Shapiro–Wilk test showed that there was a significant deviation from normality, *W*(59) = 0.94, *p* = 0.01. As such, we conducted the nonparametric Wilcoxon signed-rank test to compare the types of responses for old and new trials. On average, old trials were more frequently classified as Remember or Familiar (*M* = 38.04, *SD* = 7.97) than new trials (*M* = 5.39, *SD* = 7.08), *Z* = 6.68,* p* < 0.001, as expected.

### Influence of task demands on familiarity preferences in information-seeking

We first examined whether a familiarity preference emerged in participants’ information-seeking behaviour. Given that the type of memory judgments employed required a recall attempt, we predicted that such a preference would, again, be present. The average familiarity preference, calculated by the difference in exploration rate for previously studied versus entirely novel names, (*M* = 9.09%, *SD* = 32.91%) was significantly greater than zero, *t*(58) = 2.12, *p* = 0.04, *d* = 0.28, indicating that information-seeking was, indeed, biased towards familiar information.

### Influence of perceived recall success on subsequent information-seeking

**Figure 4:**
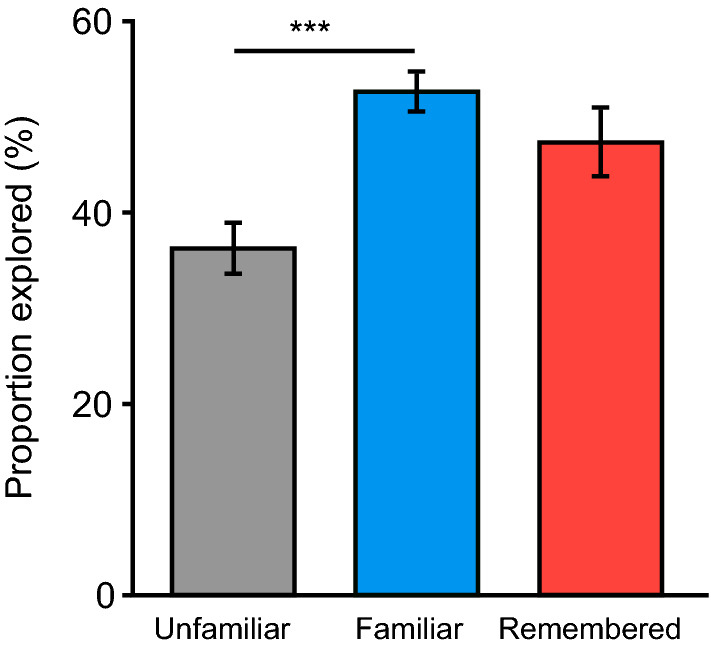
Rates of information-seeking for each of the judgement types in Experiment 2. Trials which were judged as Familiar (when the name could not be recalled) were explored more often than Unfamiliar trials (when the name was not recallable, nor the face familiar). The Remember trials (when the name was perceived to be successfully recalled) were explored more often than Unfamiliar trials. Error bars =  ± 1 SEM. ***p < 0.001.

To explore the main question of interest for Experiment 2, namely whether the familiarity preference induced by a recall attempt differed between situations in which name recall was perceived to be successful or not, we compared the frequency of subsequent information-seeking for each of our three response options. A repeated-measures ANOVA with a Greenhouse–Geisser correction revealed that there was, indeed, a significant difference across these response options (Fig. 4), *F*(1.42, 82.28) = 6.15, *p* = 0.008, η_p_^2^ = 0.096. Bonferroni-corrected post-hoc comparisons revealed, as expected based on Experiment 1, that names associated with Familiar trials (*M* = 52.60%, *SD* = 17.68%) were sought more frequently than those associated with Unfamiliar trials (*M* = 36.34%, *SD* = 22.60%), *p* < 0.001. Critically, however, they were not sought significantly more often than those associated with Remember trials (*M* = 47.26%, *SD* = 30.25%), *p* = 0.14. These post-hoc comparisons also showed a trend that Remember trials were associated with increased frequency of subsequent information-seeking relative to Unfamiliar trials, *p* = 0.07. Overall, these findings suggest that familiarity preferences in information-seeking following a recall attempt are not restricted to situations in which recall is perceived to be unsuccessful. Although other interpretations may hold, this result is compatible with the notion that familiarity preferences in information-seeking behaviour can also be driven by a motivation to validate the accuracy of information generated during a previous recall attempt.

## Experiment 3

The results of Experiment 1 demonstrate that a familiarity preference in information-seeking behaviour can be the result of a recent failed recall attempt. The results of Experiment 2 show that this preference is not limited to situations in which this recall attempt was perceived to be unsuccessful; trials in which recall of names for previously studied faces was perceived to be successful led to a comparable subsequent tendency to seek out these names as trials in which recall was unsuccessful, but the corresponding face was perceived to be familiar. We suggest that this pattern of results can be interpreted within a framework that links curiosity to the degree of uncertainty in the accuracy of information that is available to fill information gaps^9,10,28,39,41^. In Experiment 3, we sought more direct evidence in support of this account, by examining more closely how metacognitive retrieval experiences about the perceived degree of accuracy of recall are related to familiarity preferences in subsequent information-seeking behaviour.

In order to probe the perceived degree of accuracy of recall, we employed confidence judgments in combination with forced-recall instructions (e.g. Ref.^42^) that required the generation of a name on every trial, even if guessed completely (Fig. 1c). As in Experiment 1, subsequent familiarity preferences in information-seeking behaviour were probed by asking participants to choose between a previously studied versus a novel face for name exploration. Based on central claims of the RPL theory that link curiosity to the size of information gaps^29^, we expected to observe an inverse-U relationship, such that information needed to fill either large or non-existent information gaps would be sought less often than information needed to fill gaps that are close to closure (for similar results with trivia paradigms see^9,38^). In other words, we expected to see highest rates of subsequent information-seeking for previously studied faces for which recall of the associated name was expressed with a medium degree of confidence.

## Methods

### Participants

Once again, the online recruitment platform Prolific (https://prolific.co/) was used to recruit 49 English-speaking participants to participate in Experiment 3 in exchange for monetary compensation. The data of 24 participants (age range 18–33; *M* = 24.63, *SD* = 4.25) were used in all analyses. 19 participants were excluded due to an insufficient distribution of confidence ratings across the scale (see criterion from Experiment 1). 6 additional participants were excluded due to the exclusion criteria outlined in Experiment 1.

### Materials

The materials and randomization procedure for Experiment 3 were the same as those used in Experiment 2.

### Procedure

Experiment 3 involved a paradigm similar to that used in Experiment 2 (i.e. with a study phase including repeated presentations and no final forced choice recognition-memory test except for the following changes. The experiment took approximately 45 min to complete.

The most significant procedural change in Experiment 3 (Fig. 1c) related to the structure and response format of the memory-judgement trials in the second phase. As was the case in the FOK with recall condition of Experiment 1, each trial began with a self-paced recall attempt. Unlike in Experiment 1, however, participants were instructed to always provide a written answer (i.e. they were asked to engage in forced-recall^42^), whether it was a part of the name or the full name they remembered to be associated with the face. Participants were asked to guess if they were unable to remember any information. Once participants had finished typing, they proceeded to a self-paced confidence judgement, in which they were asked to rate how confident they were that the name or partial name was correct, on a 6-point Likert scale (from /‘complete guess’ to 6/‘very confident’). The next trial began after a 500 ms ISI. As before, this phase included all 52 initially studied faces, and 52 novel items for which pertinent names had never been studied. After completion of this phase, participants progressed to the exploration phase. The format of trials in the exploration phase (involving a forced-choice between a previously studied and a novel face) was the same as in Experiment 1.

## Results

### Initial validation of confidence ratings

First, we sought to confirm that the confidence ratings participants provided following their typed forced-recall attempt carried validity. To do so, we compared ratings for studied items with the ratings for new items. Given that the to-be-recalled information associated with old faces had been encountered in three study blocks but never for new faces, we expected the accompanying confidence to be higher for the former. Indeed, the average confidence associated with recall for previously studied items (*M* = 2.88, *SD* = 1.02) was significantly greater than the average confidence rating for novel ones (*M* = 1.75, *SD* = 1.15), *t*(23) = 5.59, *p* < 0.001, *d* = 1.04.

A second method we used to confirm the validity of confidence ratings involved the comparison of trials with an objectively correct recall response relative to those with an objectively incorrect response. Forced-recall responses were scored as correct if the participant typed out the correct first or last name, or both. In other words, responses were only considered incorrect if all provided information was not correct. We also accepted spelling errors if it was a slight variation, and the pronunciation would still be very similar. We expected that recalled names that were objectively correct would lead to greater feelings of subjective confidence than those that were incorrect. A Shapiro–Wilk test showed that there was a significant deviation from normality, *W*(24) = 0.89, *p* = 0.01, and as such we conducted the nonparametric Wilcoxon signed-rank test to compare the average confidence ratings. Indeed, confidence ratings were significantly higher for the names that were correctly recalled (*M* = 4.57, *SD* = 0.67) than ratings for trials that were incorrect (*M* = 1.63, *SD* = 0.47), *Z* = 4.29,* p* < 0.001. While only a minority of names were recalled correctly (*M* = 20.10%, *SD* = 18.23%) it must be noted that participants had only studied half of the face-name pairs presented in this phase. Together these results suggest that there is validity in the subjective confidence ratings provided by participants after their forced recall attempt.

### Influence of task demands on familiarity preferences in information-seeking

Before investigating the relationship between confidence and information-seeking behaviour, we examined whether we would observe increased information-seeking behaviour for names associated with previously studied as compared to novel faces following the required recall attempt. This served as a replication of the findings of the previous experiments. We conducted a Wilcoxon signed-rank test, given that there was a significant deviation from normality, *W*(24) = 0.91, *p* = 0.03. There was, once again, a significant familiarity preference (see Supplementary Materials for frequencies), as calculated by the difference in exploration frequency for previously studied versus novel information (*M* = 27.56%, *SD* = 53.31%), *Z* = 2.23,* p* = 0.03.

### Influence of subjective retrieval experiences on subsequent information-seeking

To address the primary question of interest for Experiment 3, we conducted a pair of analyses exploring, first, the relationship between confidence ratings and information-seeking choices, and secondly, between the confidence ratings and familiarity preferences. For the first of these two analyses, we rescaled the 1 to 6 confidence ratings to range from 0 to 1 and fitted Eq. (2) individually to the data from each participant.

2 $$information\, seeking\, proportion\, =\, {b}_{0}+{b}_{1}\times c +{b}_{2}\times c\times (1-c)$$

**Figure 5:**
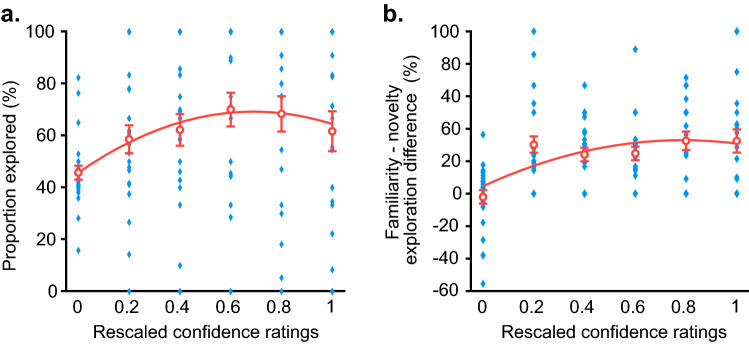
(**a**) Relationship between confidence and information-seeking behaviour in Experiment 3. The subjective confidence ratings obtained following a forced-recall attempt, had an inverse-U relationship with subsequent information-seeking behaviour. The red curve represents the equation $information\, seeking\, proportion\,=\,45.54+16.67\times c+52.97\times c\times (1-c)$, which reflects the average parameter estimates obtained when the quadratic equation was fitted to the data from individual participants. (**b**) Relationship between confidence and familiarity—novelty difference measures from Experiment 3. The familiarity—novelty difference measure was obtained at each confidence level by subtracting the number of novelty trials selected for exploration from the number of familiar trials chosen, and then dividing this difference by the total number of trials at the given confidence level. This was then multiplied by 100, to convert to a proportion. The red curve represents the equation $familiarity-novelty\, difference\, measure\,=\,4.57+26.29\times c+45.78\times c\times (1-c)$, which was, once again, obtained from the fitting of the quadratic equation to each participant's data. In both (**a**) and (**b**) the blue diamonds represent individual participant data points, and the red circles represent the means across participants. Error bars =  ± SEM.

In this equation $c$ was the rescaled confidence rating. We then conducted inferential statistics on the parameter estimates extracted from the fitted equations for each participant. This reflects the procedure in Experiment 1. On average, the model provided a ${r}^{2}$ of 0.65 (*SD* = 0.29). As predicted, there was a quadratic relationship between confidence and information-seeking, as the quadratic coefficient, $({b}_{2}$ = 49.41, *SD* = 121.2), was marginally significant, *t*(23) = 2.00, *p* = 0.058, *d* = 0.41. There was also a significant linear coefficient (${b}_{1}$= 18.76, *SD* = 43.03), *t*(23) = 2.14, *p* = 0.044, *d* = 0.44. As evident by this result (Fig. 5a), participants' tendency to seek pertinent information peaked when they had medium levels of confidence in the accuracy of the name generated in their prior recall attempt. Substantially less information-seeking occurred for items with low and high levels of confidence.

To assess whether the relationship between confidence and information-seeking motivates familiarity preferences as well, we computed a difference score that reflects familiarity preferences for each confidence level for each participant. This score was computed as in Eq. (3),

3 $$familiarity-novelty \,difference \,measure\, =\,\frac{(FR-NR)}{T}* 100\%$$

Here, $FR$ represented the number of familiar trials restudied at a given confidence level, $NR$ represented the number of novel trials restudied at the same confidence level, and $T$ represented the total number of trials at this confidence. We obtained these measures for each participant and fitted them to Eq. (4), where $c$ was, again, the rescaled confidence level.

4 $$familiarity-novelty \,difference\, measure\, = \,{b}_{0}+{b}_{1}\times c +{b}_{2}\times c\times (1-c)$$

We used inferential statistics to assess whether this preference was driven by a significant quadratic relationship. On average, the model provided a ${r}^{2}$ of 0.54 (*SD* = 0.29). Once again, results show that there was a significant quadratic coefficient (${b}_{2}$ = 45.78, *SD* = 106.8), *t*(23) = 2.10, *p* = 0.047, *d* = 0.43. As in the prior analysis, there was also a significant linear coefficient (${b}_{1}$ = 26.29, *SD* = 29.60), *t*(23) = 4.35, *p* < 0.001, *d* = 0.89. Participant’s preference to seek familiarity peaked with a confidence of 5 (Fig. 5b), which is reflected in the quadratic relationship of the fitted curve. Overall, results from these analyses demonstrate that subjective confidence is related to subsequent information-seeking and that this retrieval experience shapes the familiarity preferences we observed in this experiment.

## Discussion

The notion that novelty is a key source of curiosity, and by extension a major driver of information-seeking behaviour, has received wide empirical support in psychology and neuroscience (for reviews see Refs.^13,20,21,38^). Recent research has provided evidence, however, that familiarity preferences in information-seeking behaviour can also be observed under some circumstances, specifically when they follow a recent memory test^16^. Here, we conducted three experiments to identify critical retrieval factors in memory tasks that may induce a subsequent familiarity preference in information-seeking. In Experiment 1 we demonstrated the critical role of an explicit recent unsuccessful recall attempt in inducing a subsequent familiarity preference for information that could not be recalled. The results of Experiment 1 did not provide support for the suggestion that making predictions about future performance at the time of recall is a critical factor; FOK judgments that required such predictions induced a comparable preference as familiarity judgments that did not. Moreover, retrieval experiences for both types of judgements predicted corresponding preferences in subsequent information-seeking. Results of Experiment 2 showed that the impact of recent recall attempts on subsequent familiarity preferences is not limited to situations in which recall is perceived to be unsuccessful; we also observed such preferences in information-seeking following trials that were associated with subjectively perceived recall success. In our final experiment, we showed that confidence in the accuracy of the information generated during recall is a key factor, with medium degrees of confidence leading to the highest subsequent familiarity preference in information-seeking. Overall, these findings provide new evidence in support of the idea that novelty preferences in information-seeking behaviour are not ubiquitous, and that familiarity can trump novelty when this behaviour follows a recent related retrieval attempt. Our results suggest that specific task demands of retrieval, as well as metacognitive retrieval experiences, are important determinants of such familiarity preferences.

The results of the current study can be understood within theoretical frameworks of state curiosity that emphasize its role in motivating information-seeking so as to fill information gaps^2,29^. Recall attempts and corresponding metacognitive retrieval experiences are thought to play a critical role in the identification and the assessment of the size of such information gaps; in turn they can provide valuable guidance for their closure. Of particular relevance, the RPL theory suggests that when unsuccessful recall attempts point to existing gaps in knowledge, subsequent curiosity is highest for information that most easily allows for closure of a gap, as determined based on metacognitive retrieval experiences^26–29^. While we did not set out to test the RPL framework directly, it does allow for interpretation of our findings on familiarity preferences in information-seeking in a theoretically guided way.

Experiment 1 revealed that the explicit requirement for a recall attempt in a memory test boosts subsequent preferences for familiar information when compared to a test in which this requirement is not present. This finding is of particular importance for the assessment of familiarity, given such judgments may be less likely than FOK judgments to include a spontaneous recall attempt. According to the RPL framework, an unsuccessful recall attempt is critical for inducing curiosity as the outcome of this attempt offers evidence as to whether an information gap exists^29^. Indeed, on the large majority of trials in Experiment 1, attempted recall was ultimately deemed unsuccessful. In Experiment 2, however, we demonstrated that familiarity preferences after a recall attempt do not only occur when recall is perceived to be unsuccessful but can also be observed when there is subjectively perceived success. At first glance, this result may appear to conflict with the notion that familiarity preferences are tied to recently experienced information gaps^43^. We suggest, however, that it can still be interpreted with reference to such gaps if one considers that familiarity preferences may also reflect a tendency to seek feedback about the accuracy of the information generated in a recent recall attempt. From this perspective, the degree of uncertainty in the answer that was generated during recall marks the size of the perceived information gap, with answers accompanied by relatively higher uncertainty corresponding to larger perceived gaps in one’s knowledge. Indeed, in a recent study that probed determinants of curiosity in the trivia paradigm, Singh and Manjaly reported that curiosity and information-seeking behaviour increase in direct response to the degree of uncertainty, even when the amount of missing information is the same^39^. Experiment 3 of the current study revealed evidence in support of the notion that familiarity preferences in information-seeking may also be tied to uncertainty in the behavioural paradigm we employed. Specifically, we observed an inverse U-shaped relationship between confidence in answers that were generated during forced-recall attempts and the likelihood of subsequently engaging in pertinent information-seeking behaviour.

Across experiments we demonstrated that familiarity preferences in information-seeking are related to different kinds of metacognitive retrieval experiences (FOK, familiarity, and confidence). In Experiment 1, higher FOK and higher familiarity ratings were associated with greater subsequent information-seeking for familiar information. In Experiment 3, this preference was most pronounced for information recalled with medium confidence. Each of these metacognitive retrieval experiences may be understood as an estimate of the size of an information gap. From this perspective, our results are generally compatible with the core notion of RPL, namely that curiosity peaks when an information gap is of an optimal size (i.e. the range in which it is small enough to be judged as possible to be closed). They also suggest, however, that this peak may differ for different kinds of retrieval experiences.

Why would the strongest familiarity preference in information-seeking be associated with medium levels of recall confidence but with the highest levels of FOKs and feelings of familiarity? One possibility is that different encoding conditions across experiments led to differences in memory performance for face-name associations, which in turn led to differences in the size of perceived information gaps. A comparison of our findings for Experiment 2 versus Experiment 1 suggests that the introduction of multiple study blocks lead to the emergence of more trials in which recall of names was perceived as successful. Inasmuch as Experiment 3 also employed multiple study blocks, it is likely that trials with high confidence in recall were associated with no perceived information gap, while medium confidence would characterise situations with an optimally-sized information gap. By contrast, the single study block included in Experiment 1 led to a very small number of trials with perceived success in recall when directly probed. As such, most trials with high FOKs and those with strong feelings of familiarity can be expected to be associated with a notable information gap based on the outcome of the associated failed recall attempts.

It should be noted that recall confidence, FOK experiences, and feelings of familiarity are all distinct in their phenomenological characteristics as well (for reviews see^37,44,45^). These differences in phenomenology may be tied to the described differences in estimation of the size of information gaps, and, in turn, to the degree of curiosity that they generate. An important distinction in this context is that confidence, as probed in Experiment 3, is a metacognitive experience about information that was generated successfully during a recall attempt whereas FOKs, as probed in Experiment 1, reflect metacognitive experiences about (future) recognition of information, typically in association with failed recall^37^. Similarly, there are important characteristics that differ between FOKs and familiarity (e.g. Refs.^37,46^). Familiarity judgments, as administered in Experiment 1, require reflection on the exposure history of the stimulus itself (i.e. faces in this case). FOK judgments, by contrast, require reflection on information that was associated with such stimuli (i.e. their names). This difference in task demands may explain why the relationship of judged familiarity to subsequent information-seeking was mathematically best captured with the introduction of a quadratic term, whereas a linear term was sufficient to capture this relationship for the FOK conditions in Experiment 1. The slight increase in information-seeking for stimuli perceived to be novel as compared to moderately familiar, which is reflected in this quadratic component, may reflect the type of novelty preference that has been observed in many prior studies (as reviewed in the Introduction). Regardless, our observation that the highest levels of familiarity were associated with the numerically highest rates of subsequent information-seeking converges with the results from Experiment 2 showing, based on use of categorical responses, that items judged to be familiar tend to be sought out more frequently than items judged to be novel when critical associated information cannot be recalled.

A second task factor we examined in relation to familiarity preferences was whether the memory task required making predictions about future performance. We considered this factor based on work in the educational literature showing that the act of making predictions about the filling of information gaps can increase curiosity^30–33^. To get at this issue within the context of the current behavioural paradigm, we manipulated the nature of the memory task employed in the retrieval phase of Experiment 1. Specifically, we compared familiarity preferences after FOK judgements and familiarity judgments, which did or did not require predictions about future performance, respectively. Our results revealed that subsequent familiarity preferences in information-seeking were not affected by this task manipulation. Instead, we found that variations in graded retrieval experiences for both types of memory judgements showed a comparable relationship to subsequent familiarity preferences. Critically, effects of predictions on curiosity in educational research have been interpreted with reference to the role such predictions may have in highlighting pertinent knowledge gaps^31^. We have argued that, in the current experimental set-up, even the memory-test conditions that do not require making predictions, such as familiarity judgments, can trigger subjective awareness of gaps in knowledge. The most relevant evidence in support comes from our results from the condition in which recall was probed in combination with familiarity judgments in Experiment 1. Here, where the explicit recall attempt was deemed unsuccessful on the large majority of trials, we demonstrated that a clear preference for familiar information emerged and was related to the size of the identified information gap. Taken together these findings suggest that whether making predictions increases curiosity depends on whether other situational factors are already at play that could highlight knowledge gaps.

Finally, we note that in the current study we employed two distinct task formats to examine familiarity preferences in the exploration phase that offered access to select names. In Experiments 1 and 3, pairs of faces were presented on each trial and participants were required to choose between a familiar face, whose name they had initially studied, and a novel one, whose name they had not seen before. By contrast, in Experiment 2, participants were asked to make choices for individually presented previously studied and novel faces under conditions in which the number of trials for which information about the corresponding name could be sought was limited. That we observed familiarity preferences that were shaped by retrieval experiences during a recent memory test in both exploration formats demonstrates the robustness of this phenomenon. As such these results provide additional support for our broader conclusion that novelty preferences in information-seeking behaviour are not ubiquitous. Collectively, our findings argue in favour of the view that familiarity can trump novelty when information-seeking behaviour follows a retrieval situation in which a sizable gap about previously learned information was identified.

## Supplementary Information

Supplementary Information.

## Data Availability

The datasets generated during and/or analysed during the current study are available from the corresponding author on reasonable request.
