# Supplementary material for: When familiarity not novelty motivates information-seeking behaviour
Source: Sci Rep. 2023 Mar 30;13:5201. doi: 10.1038/s41598-023-31953-6 (PMC10063652; doi:10.1038/s41598-023-31953-6)
Supplement: Supplementary file 1 — Supplementary Information. [file 41598_2023_31953_MOESM1_ESM.pdf]

When familiarity not novelty motivates information-seeking behaviour

Gregory Brooks, Hannah Whitehead, Stefan Köhler

Supplementary Material

Experiment 1

Results

Validity of FOK ratings. FOK-rating validity is typically assessed through correlations between the rating and subsequent recognition-memory accuracy. In our current experimental set-up, we introduced an Exploration phase between when we elicit FOK ratings and when the recognition-memory test takes place, along with novel items. Due to this manipulation, we computed correlations between FOK rating and subsequent recognition-memory accuracy separately for items that were not sought and those that were sought, always restricting it items that had been studied initially. In the FOK with recall condition the correlation for not sought trials (*mean* γ = 0.08, *SD* = 0.41) was not significant, *t*(27) = 1.03, *p* = 0.31, *d* = 0.19, but was for trials that were sought (*mean* γ = 0.27, *SD* = 0.28), *t*(27) = 5.15, $p < 0.001$ *d* = 0.97. In the FOK no recall condition, both trials that were not sought (*mean* γ = 0.17, *SD* = 0.34) and those that were (*mean* γ = 0.28, *SD* = 0.33) were associated with significantly positive correlations, *t*(27) = 2.69, *p* = 0.01, *d* = 0.51 and *t*(26) = 4.37, $p < 0.001$, *d* = 0.84, respectively. These findings generally support the validity of FOKs, especially when combined with the finding of higher FOK ratings for old compared to new items, as reported in the main manuscript.

Influence of retrieval-task demands on familiarity preferences in subsequent information-seeking. In Experiment 1 we report the data on familiarity preference as the difference in exploration frequency between previously studies and novel items. In the FOK with recall condition, old trials were sought on 57.55% (10.70) of the trials. In the familiarity with recall condition old trials were sought at a rate 57.01% (6.52). In the FOK no recall condition old trials were sought at a frequency of 54.40% (12.14). Finally, in the familiarity no recall condition old trials were chosen at a rate of 51.92% (6.95). The exploration frequency for new items is equal to 100 – the old item exploration rate, with the same standard deviation. This is due to the experimental design of the exploration phase, with a side-by-side choice between old and new.

Influence of retrieval experiences on subsequent information-seeking. We repeated the analyses from Experiment 1 that assessed the relationship between retrieval experiences and subsequent information-seeking choices, but with the inclusion of all items regardless of objective old-new status. Once again, in each of our conditions, a significantly positive correlation was observed (FOK with recall: *mean* γ = 0.21, *SD* = 0.22, *t*(27) = 4.97, $p < 0.001$, *d* = 0.94; FOK no recall: *mean* γ = 0.15, *SD* = 0.24, *t*(27) = 3.17, *p* = 0.004, *d* = 0.60; Familiarity with recall: *mean* γ = 0.14, *SD* = 0.20, *t*(27) = 3.71, $p < 0.001$, *d* = 0.70; Familiarity no recall: mean γ = 0.08, *SD* = 0.20, *t*(27) = 2.18, *p* = 0.04, *d* = 0.41). Additionally, there was no difference in this relationship between conditions, *F*(3,108) = 1.64, *p* = 0.19. These findings align with

those reported in the main manuscript. They demonstrate that the links between retrieval experiences and information-seeking are not limited to FOK and extend to the subjectively experienced degree of familiarity with the stimulus itself.

Experiment 2

Supplementary Table S1.

Summary of memory-judgement response frequency in Experiment 2 according to objective status.

	Remember		Familiar		Unfamiliar	
	**Number**	**Proportion (%)**	**Number**	**Proportion (%)**	**Number**	**Proportion (%)**
**Previously Studied**	12.81 (8.23)	12.32	25.22 (7.75)	24.25	13.97 (7.97)	13.43
**Novel**	0.51 (1.83)	0.49	4.88 (5.66)	4.69	46.61 (7.08)	44.82

Notes: Data are shown as Mean (SD).

Total trial number is 104 (52 previously studied, 52 novel).

All proportions reported add to 100%.

Experiment 3

Results

Influence of task demands on familiarity preferences in information-seeking. In Experiment 3, objectively novel items were sought, on average, at a frequency of 36.22% (*SD* = 26.75%). This value entered our computation of the difference measure for familiarity preferences reported in the main manuscript.
